# A New Strategy for the Detection of Chicken Infectious Anemia Virus Contamination in Attenuated Live Vaccine by Droplet Digital PCR

**DOI:** 10.1155/2019/2750472

**Published:** 2019-05-16

**Authors:** Qiuchen Li, Yubiao Zhang, Fanfeng Meng, Hui Jiang, Guanlong Xu, Jiabo Ding, Yawen Zhang, Guiwei Dong, Sibao Tian, Shuang Chang, Peng Zhao

**Affiliations:** ^1^College of Veterinary Medicine, Shandong Agricultural University, Tai'an, Shandong, China; ^2^Shandong Provincial Key Laboratory of Animal Biotechnology and Disease Control and Prevention, Tai'an, Shandong, China; ^3^Shandong Provincial Engineering Technology Research Center of Animal Disease Control and Prevention, Tai'an, Shandong, China; ^4^Yuyao Animal Husbandry and Veterinary Bureau, Ningbo, Zhejiang, China; ^5^Beijing Dafaun Poultry Breeding Company Ltd., Beijing, China; ^6^National Reference Laboratory for Animal Brucellosis, China Institute of Veterinary Drug Control, Beijing 102629, China

## Abstract

Chicken infectious anemia virus (*CIAV*) causes the atrophy of bone marrow hematopoietic and lymphoid tissues in chicks, leading to huge economic losses all over the world. The using of attenuated vaccine contaminated with CIAV increased the mortality and the pathogenicity of other diseases in many farms. However, it is difficult to detect the CIAV contamination by general detection technology due to the extremely low dose of CIAV in vaccines. In this study, we established a new method called droplet digital Polymerase Chain Reaction (*ddPCR*) to detect CIAV contamination of vaccines more sensitively and accurately. The lowest detection limitation of this method is 2.4 copies of CIAV plasmid or CIAV contamination at 0.1 EID_50_/1000 feathers in vaccines without any positive signals of other viruses. Besides, the sensitivity of ddPCR is 100 times greater than that of conventional PCR and 10 times greater than that of real-time PCR. The ddPCR technique is more sensitive and more intuitive. Therefore, it could be valuable for the detection of CIAV contamination in vaccines.

## 1. Introduction

Chicken infectious anemia (CIA) is an economically important disease affecting the poultry industry worldwide which is caused by chicken infectious anemia virus (*CIAV*) [1, 2]. CIAV causes chicken systemic lymphoid tissue atrophy, especially bone marrow hematopoietic tissue and lymphoid tissue, leading to immunosuppression. Up to now, the prevalence of CIAV has caused huge economic losses all over the world [1–6]. Chickens in different ages can be infected by CIAV, but the clinical symptoms mainly appear in 10 to 14 days old [6]. CIAV mainly causes increased mortality, growth retardation, anemia, bone marrow regenerative disorders, and thymus atrophy [7]. The disease appeared early in small-scale poultry farms that lacked favorable biological safety conditions and then erupted in some large-scale modern poultry farms [8]. Recently, the positive rate of CIAV antibody in chickens of China has continued to increase, especially in some local breeds [2, 9]. CIAV can be transmitted vertically and horizontally but vaccines contaminated with CIAV were also suspected as one of the sources of infection in recent years [10].

In past 20 years, there were many exogenous viruses which were detected in attenuated vaccines, while Avian leucosis virus (*ALV*) occupied a main position, followed by reticuloendotheliosis virus (*REV*), CIAV, and fowl adenovirus (*FADV*) [10–14]. Because of the particularity of the vaccination, once contaminated attenuated vaccine of exogenous virus is used, the disease would break out in the affected poultry farm which would lead to great economic losses. Therefore, strengthening the monitoring of attenuated vaccines contaminated with CIAV is very urgent for the prevention and control of CIAV infections in large-scale breeder chicken flock. At present, the detection of CIAV in biological products, such as Marek's disease virus vaccines, is mainly based on the results of specific pathogen free (*SPF*) chicken tests. This test requires a long time and a strictly regulated experimental animal facility. Molecular detection method, such as PCR, was used to detect CIAV. Generally, the dose of CIAV and other exogenous viruses in live vaccines is very low [8, 10–14]; therefore it is necessary to establish a sensitive and specific detection method. Since the sensitivity and specificity of PCR are not satisfactory, a modification is needed.

Droplet digital PCR (*ddPCR*) is a new absolutely quantitative technique for nucleic acid [15]. In the process, a standard PCR reaction system is dispersed into a certain volume droplets, and each microdroplet will contain 1 or 0 copies nucleic acid [16]. After then, ddPCR is carried out and the number of positive or negative above droplets will be determined based on fluorescence signal [17]. The copy number of nucleic acids in the sample was calculated by the statistical method of Poisson distribution. In this study, a digital PCR method for CIAV detection has been established in order to provide a more accurate technique for the contamination of exogenous viruses in vaccines.

## 2. Materials and Methods

### 2.1. Virus and Vaccine

Specific-pathogens-free (SPF) chick embryos were purchased from Jinan SPAFAS Poultry Co. and CIAV SDLY08 strain (Genbank No. FJ172347.1) was isolated from a commercial broiler in Shandong. The liver (stored in our lab) infected with CIAV was grinded and filtered with 0.22*μ*m filters. The solution was then diluted (10^−1^-10^−7^), and 0.1 mL aliquots of each dilution were used to inoculate six 7-day-old SPF chicken embryos through the yolk sac. The embryos died within the next 24 hours were excluded. CIAV infection of the remaining embryos was detected in 18-19 days of age. The 50% Egg Infectious Dose (*EID*_50_) of the strain (10^5^ EID_50_/ml) was calculated according to the Reed-Muench method. The common Newcastle disease virus attenuated vaccine (*NDV*), Fowlpox virus vaccine (*FPV*), and infectious bronchitis virus vaccine (*IBV*) were purchased from Qingdao Yebio Bioengineering Co., Ltd., and have been identified by China Institute of Veterinary Drug Control as qualified products that do not contain any exogenous virus. And the experiments were performed in a Biosafety Lab II in China Institute of Veterinary Drug Control.

### 2.2. Primers and Probes Design

Based on the published sequences of CIAV from NCBI, lasergene 7.0 was used to identify conserved regions in them. Primers and probes for CIAV were designed to run specific PCR, qPCR, and dd-PCR (Table 1). FAM was used as the fluorescence reporter group and BHQ as the fluorescence quenching group. The primers and probes were synthesized and labeled by Shanghai Sangon Biotech Co., Ltd.

### 2.3. Preparation of Recombinant Plasmid

The size of the targeted fragments in the* VP2* gene of SDLY08 was 164 bp and the amplification system was 50 *μ*L with the following PCR conditions: initial incubation 95°C for 5 min, followed by 31 cycles of denaturation at 95°C for 30 s, annealing at 55°C for 30s, extension at 72°C for 30 s, and a final extension at 72°C for 10 min. PCR products were obtained by agarose gel electrophoresis and the target bands were cloned and sequenced. The correct sequence plasmid was designated PMD18-T-CIAV. The positive standard product was quantified using a nucleic acid quantizer. The plasmid copy number of PMD18-T-CIAV was calculated as follows: [Copies/ml=6.02*∗*10^23^(copies/mol)*∗*concentration of plasmid(g/ml)/MW(g/mol)] and serial 10-fold dilutions were prepared as the template and stored at −20°C.

### 2.4. Optimization of PCR, qPCR, and ddPCR

The method of conventional PCR and qPCR to detect CIAV was established and optimized. The PCR conditions were as follows: denaturation for 5 min at 95°C, followed by 31 cycles of 95°C for 30s, 55°C for 30s, and 72°C for 30s, with a final extension at 72°C for 10 min. Then the PCR amplification products were analyzed by 1.0% agarose gel electrophoresis. A qPCR assay was developed for detecting CIAV with the primers probe using an ABI 7500 Real-Time PCR System (USA). The optimal reaction conditions, which were established by using the matrix method, were as follows: a 20 *μ*L total volume containing 10 *μ*L of 2×Premix Ex Taq (RR064A, TAKARA), 0.5*μ*L (10*μ*mol/L) of the F and R primer, 0.8*μ*L (25*μ*mol/L) of probe, 2 *μ*L of template, and 6.2*μ*L of H_2_O with the following PCR conditions: predenaturation at 95°C for 30s followed by 40 cycles of denaturation at 95°C for 5s and annealing at 60°C for 34s. The ddPCR reaction system was established including the preparation system, microdroplet generation, amplification cycle, signal reading, and specific operation. Different concentrations of primers and probes were used to optimize the reaction and explore the optimal annealing temperature. The optimized ddPCR conditions were 20 *μ*L of 10 *μ*L 2×mix (1863010, BIO-RAD, USA), 1.8 *μ*L (10*μ*mol/L) of the upstream and downstream primers, 0.5 *μ*L of probe (25 *μ*mol/L), 2.4 *μ*L of template, and 3.5 *μ*L of H_2_O. The reaction conditions consisted of a 10 min predenaturation at 95°C, followed by 40 cycles of 20 s at 95°C and 40 s at 58°C, with a final step of solidification for 10 min at 98°C. The rate of temperature increase and decrease was 2.5°C/s. The signal was read and analyzed immediately after ddPCR. The positive copies concentration was calculated as follows: concentration= [-ln (negative droplets/total droplets)]/droplet volume.

### 2.5. Specificity, Sensitivity, and Reliability of PCR, qPCR, and ddPCR

The specificity of the three methods was verified using NDV vaccines, FPV vaccines, and IBV vaccines. Genomic DNA of FPV was extracted from vaccines using a DNA extraction kit (D3892, Omega Bio-Tek, Norcross, GA, USA). Meanwhile, the RNA of NDV and IBV were extracted using an RNA extraction kit according to the manufacturer's instructions (R6874, Omega Bio-Tek), and then reverse transcripted into cDNA using a reverse transcription kit (6210A, Takara). The sensitivity of PCR, qPCR, and ddPCR was evaluated by 10-fold gradient dilution of recombinant plasmid from 10^3^ to one copy per microlitre. In order to evaluate the repeatability and stability of those methods, inter- and intrarepeated tests were conducted in triplicate, respectively.

### 2.6. Application of PCR, qPCR, and ddPCR to Detect CIAV Contamination in Vaccines

The quantified SDLY08 was diluted to 100 EID_50_/mL, 10 EID_50_/mL, 1 EID_50_/mL, and 0.1 EID_50_/mL with sterile PBS. Four bottles of NDV vaccine (1,000 feathers) from the same batch were diluted using the PBS solution that contained SDLY08 in different titers. The total DNA was extracted as the template according to the operation instructions of the DNA Extraction Kit (OMEGA). The ddPCR method established in this study was used to detect CIAV contamination with different gradients in the vaccine. Additionally, the same test was performed using conventional PCR and qPCR methods, respectively.

## 3. Results

### 3.1. Preparation of Standard Samples

**Figure 1:**
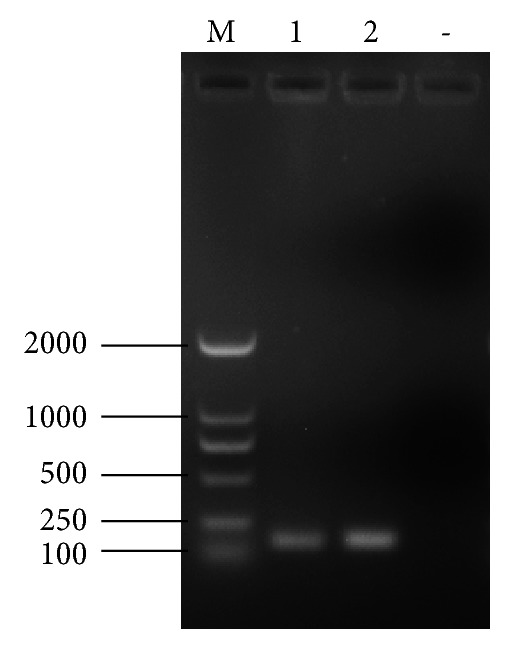
Electrophoretic result of the CIAV target fragment. A target fragment of approximately 164 bp was obtained in the two repetitions (1,2).

As illustrated in Figure 1, a target fragment of 164 bp was obtained using the primers designed for ddPCR. Sequencing of the recombinant plasmid revealed a 100% homology between the inserted gene and the reference sequence, indicating that recombinant plasmid was successfully constructed. The concentration of plasmid was measured by an ultramicrospectrophotometer and then determined as 310 ng/*μ*L, which is equivalent to 1.01 × 10^11^ copies/*μ*L. Then it was serially 10-fold diluted from 10^11^ to 10^0^ copy/*μ*L.

### 3.2. Sensitivity, Specificity, and Repeatability of PCR, qPCR, and ddPCR Detection of Standard Recombinant Plasmid

**Figure 2:**
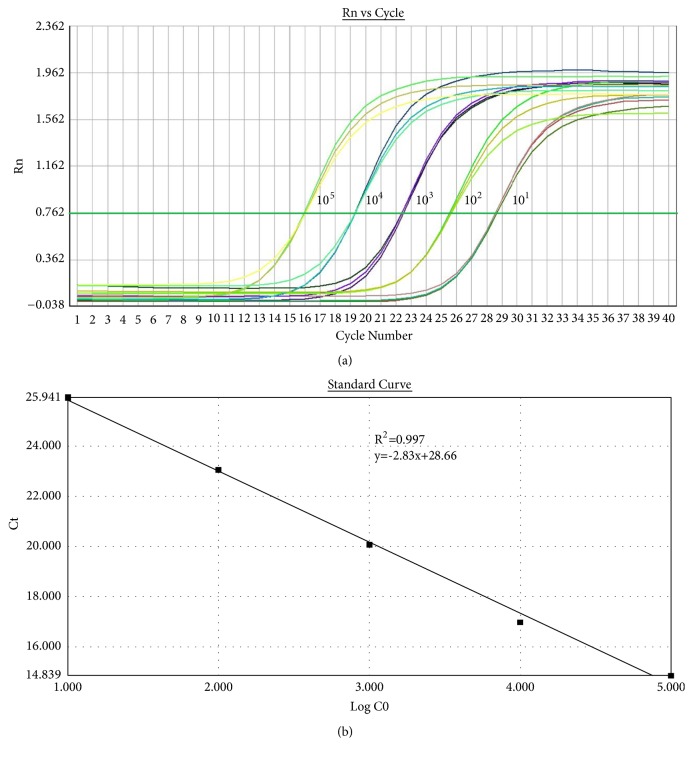
Standard curve of CIAV by qPCR.

The serially diluted CIAV standard plasmids were used as the template to verify the sensitivity and repeatability of these methods while cDNA samples from the NDV, IBV, and FPV vaccines were used to verify the specificity (Table 2). The positive target band could be observed using conventional PCR method only when the concentrate of plasmid was higher than 10^2^ copies/*μ*L, indicating the lowest limitation of standard recombinant plasmid was 10^2^ copies/*μ*L. And no signal could be detected while NDV, IBV, and FPV samples were used as the template. The kinetic and standard curves of CIAV were obtained while using qPCR method. Results showed that there was a linear relationship between 10^5^ and 10^1^ copies/*μ*L, with an R^2^ value of 0.997, indicating that the lowest limitation of this method is 20 copies per reaction (Figure 2). The coefficient of variation (*CV*) for the intra- and intergroup replicates ranged from 0.22 to 0.89%, which indicates the method is accurate and has good reproducibility. The specificity of the qPCR was assessed using cDNA of NDV, IBV, and FPV vaccines, showing that only poor signals were obtained for other viral nucleic acids.

**Figure 3:**
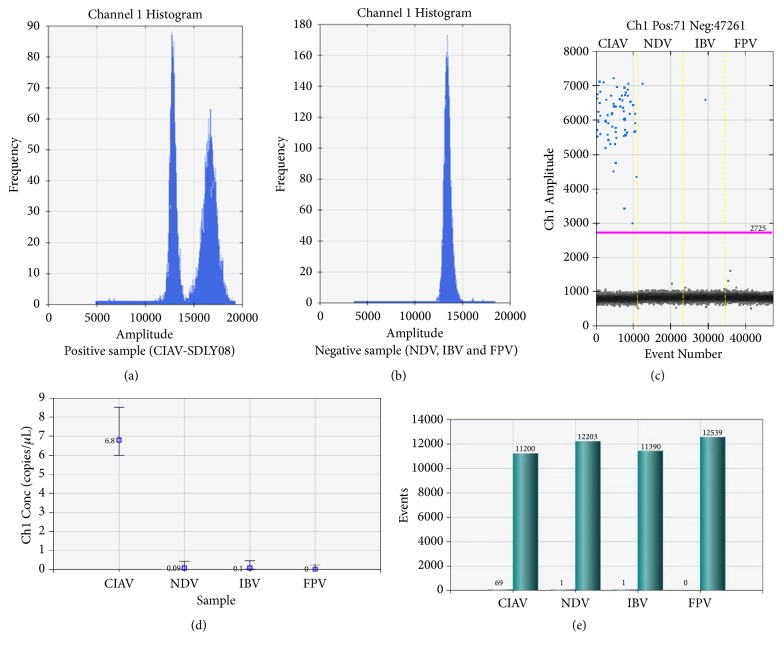
The determination of ddPCR specificity. (a) Peak frequency of positive droplets detected CIAV-SDLY08. (b) Peak frequency of negative droplets detected NDV, IBV, and FPV. (c) The ddPCR results for CIAV, NDV, IBV, and FPV detection. The unbroken pink line is the threshold, above which are positive droplets (blue) with PCR amplification and below which are negative droplets (gray) without any amplification. Four ddPCR reactions with different virus of template are divided by the vertical dotted yellow line. (d) Copy numbers of ddPCR results for CIAV, NDV, IBV, and FPV. (e) The number of positive droplets in CIAV, NDV, IBV, and FPV samples.

**Figure 4:**
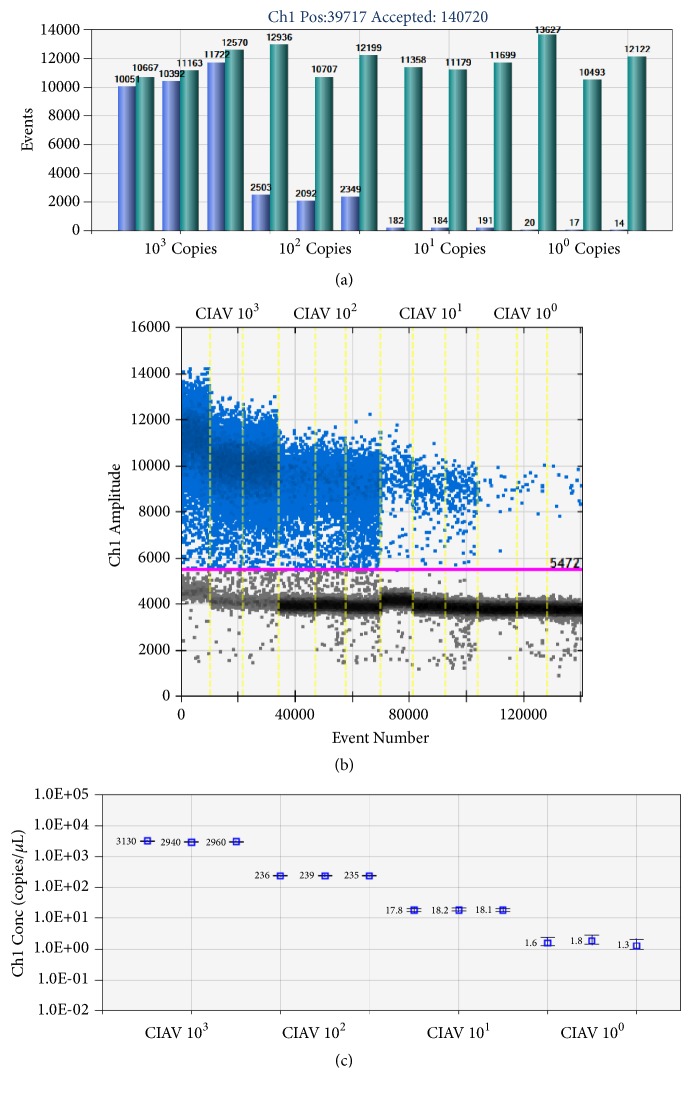
dd-PCR with standard plasmid. (a) Positive and total droplets in tenfold serial dilution series of positive plasmid DNA. (b) Results of droplet amplification with different concentrations of the standard samples. (c) Linear regression of the ddPCR assay for the copy number of samples with different concentrations. The vertical axis shows the log 10 -transformed copy number/*μ*L of the ddPCR reaction mixture. The horizontal axis indicates tenfold serial dilution series of positive plasmid DNA of the ddPCR reaction. The inner error bars indicate the Poisson 95% confidence interval (CI) and the outer error bars show the total 95% CI of replicates.

The ddPCR using NDV vaccine contaminated with CIAV-SDLY08 at a dose of 1 EID_50_ produced double peaks: peaks of negative droplets and peaks of positive droplets (Figure 3(a)) while there was only a single negative droplet peak for other pathogens (Figure 3(b)), such as NDV, IBV, and FPV, indicating the specificity of the method is pretty good. In the negative samples, only one or zero positive droplet was detected (no more than 0.1 copies/*μ*L) showing that the ddPCR has good specificity to detect CIAV without false positives (Figures 3(c), 3(d), and 3(e)). As shown in Figure 4(a), at least 10000 microdroplets were generated in every sample. What is more, ddPCR has a very high sensitivity and could accurately detect 2.4 copies of standard plasmid; besides, it showed a good linear relationship with the increase of the concentration of template from 1 to 10^3^ copies/*μ*L (Figures 4(b) and 4(c)). Reproducibility testing showed that when the quantity exceeded 2.4 copies, it could be detected stably with an inter- and intra-branch coefficient of variation (*CV*)< 4%, which indicated this method had good repeatability and could be tested stably and reliably (Table 3).

### 3.3. Comparison of Detection Sensitivity of CIAV in Vaccine among ddPCR, qPCR, and PCR

**Figure 5:**
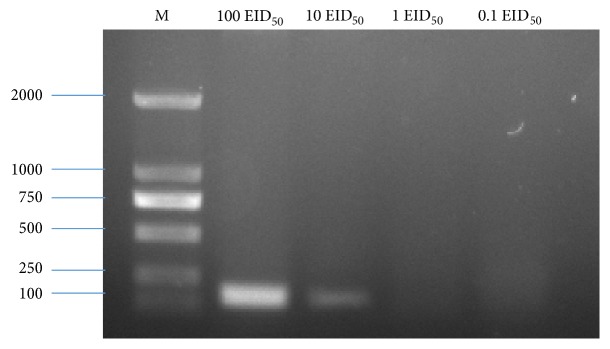
The sensitivity of routine PCR for detecting different dose of CIAV contamination in vaccines. Routine PCR tests of vaccines contaminated with different doses of CIAV showed a positive result at 10 EID_50_ for each vaccine and a negative result below this dose.

**Figure 6:**
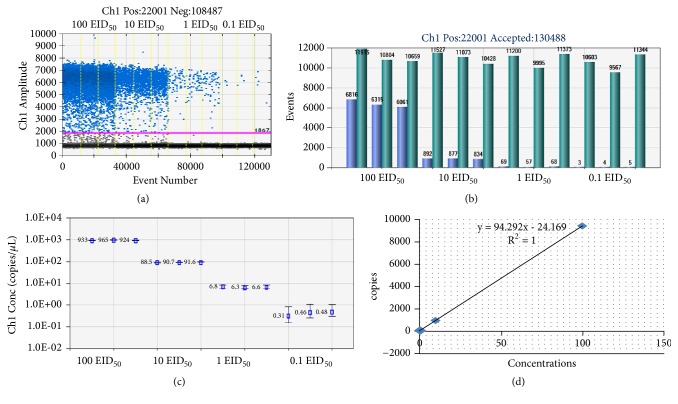
dd-PCR with vaccines contaminated with CIAV. (a) Results of droplet amplification with different concentrations of contaminating CIAV. The unbroken pink line is the threshold, above which are positive droplets (blue) with PCR amplification and below which are negative droplets (gray) without any amplification. (b) Positive and total droplets in the samples with different amount of contaminating CIAV. Low light blue is the number of positive droplets. High dark green is the number of total droplets. (c) Linear regression of the ddPCR assay for the copy number in vaccine contaminated with different dose of CIAV. The vertical axis shows the log 10 -transformed copy number/*μ*L of the ddPCR reaction mixture. The horizontal axis indicates tenfold serial dilution series of the CIAV-contamination (100, 10, 1, and 0.1 EID_50_) of the ddPCR reaction. The inner error bars indicate the Poisson 95% confidence interval (CI) and the outer error bars show the total 95% CI of replicates. (d) The standard curve of the ddPCR with different concentrations. The estimated Pearson correlation coefficient of the CIAV copy regression curve (y = 94.293x—24.169) is 1 (R^2^ = 1, P< 0.001).

CIAV at different doses (100, 10, 1, and 0.1 EID_50_) were added into NDV vaccines and then the total DNA of the vaccine was extracted. When conventional PCR was used to detect the CIAV in the vaccine, the positive target band could be observed only when the vaccine was contaminated by 10 EID_50_ CIAV per bottle or higher, while no signal could be observed if the contamination dose was 1 or 0.1 EID_50_ (Figure 5). The qPCR assay was able to detect CIAV contamination of 100, 10, and 1 but 0.1 EID_50_ (Table 4). When the contamination dose was at 0.1 EID_50_, the reproducibility of qPCR was poor and the CT value was very close to the negative control. The ddPCR showed that the amount of CIAV exceeded 0.1 EID_50_, with more viral copies detected as the contamination increased (Figures 6(a) and 6(b)). In vaccines contaminated with CIAV, even at a dose of only 0.1 EID_50_, calculated copies number was, respectively, 0.31, 0.46, and 0.48 copies/*μ*L in the intragroup repeats (3, 4, and 5 positive droplets could be observed in Figure 6(a)). The copies number was significantly higher than that of negative control group (0.09, 0.1, 0) (p < 0.05) and it could be detected stably with CV <50% (Table 5), which suggested it has a good repeatability. What is more, there is a linear relationship between the contamination dose from 0.1 to 100 EID_50_ and the number of copies detected (Figures 6(c) and 6(d)). Thus, the lowest limitation of ddPCR was 0.1 EID_50_ contamination of CIAV in vaccine. Therefore, above results demonstrated that the sensitivity of ddPCR is 100 times higher than that of conventional PCR and 10 times higher than that of qPCR.

## 4. Discussion

In 1979, CIAV was first isolated from a vaccine [18]. Many experts believe that the universal transmission of CIAV in chickens is related to the contamination in attenuated vaccines [8], which even have been reported more frequently in recent years. In 2018, it has been reported that the NDV attenuated vaccine cocontaminated with FAdV and CIAV causes inclusion body hepatitis-hydropericardium syndrome in poultry, while CIAV increased the mortality of HPS-infected birds [8]. What is more, two exogenous CIAV strains were also isolated and identified from a total of 14 batches of live-virus vaccines by PCR [10], even though its sensitivity was limited, which highlighted the need for screening contamination in live vaccines. At present, main method used for detecting CIAV is virus isolation and identification, electron microscopy, agar diffusion assay, ELISA, PCR, and qPCR, but all these methods have some drawbacks. Since the dose of CIAV contamination in vaccine is usually extremely low, common detection methods such as PCR are ill-suited for detection due to its low sensitivity. Moreover, serum neutralization and indirect immunofluorescence tests are time-consuming and laborious. Therefore, it is necessary to establish a fast, simple, and sensitive diagnostic method to detect the CIAV contamination in vaccines.

In recent years, ddPCR has been developed as an alternative to real-time quantitative PCR [16]. Compared with qPCR, the most obvious advantage of ddPCR is that it does not need a standard curve and can directly obtain the copy number of the targeted exogenous virus in the sample, which is an absolute quantity of the initial sample. What is more, in Dong's research, the ddPCR assay established showed higher sensitivity than qPCR in the detection of FAdV-4 contamination in vaccines, which suggested ddPCR is a viable method for virus detection and quantification [19]. In our study, the system of qPCR and ddPCR was optimized using the matrix method. Different concentrations of primers and probe as well as various annealing temperatures were tested. In the optimized system, even 2.4 copies of CIAV plasmid in the system could be detected stably by ddPCR while the lowest detection limitation of qPCR is 20 copies, suggesting the higher sensitivity of ddPCR. With its simple and reliable characteristics, ddPCR has potentially broad applications for the quantitative detection of DNA virus and has been used for the detection of virus, zoonotic bacterial pathogens, waterborne RNA viruses, and low-concentration host messenger RNA transcripts [20–23]. Moreover, the ddPCR technology established in this study provides a new method for detecting the low-dose contamination of CIAV in the attenuated vaccine. Using ddPCR, less than one droplet (calculated copy numbers less than 0.1 copies/*μ*L) was detected in the NDV, IBV, and FPV vaccines. In vaccines contaminated with CIAV, even at a dose of only 0.1 EID_50_, calculated copies number was, respectively, 0.31, 0.46, and 0.48 copies/*μ*L in the intragroup repeats (3, 4, and 5 positive droplets could be observed). The copies number was significantly higher than that of negative control group (p < 0.05) and it could be detected stably with CV <50%, which suggested it has a good repeatability. There was a linear relationship between the amount of contamination and the number of copies. On the contrary, the method of qPCR can only detect the contamination at 1 EID_50._ When the contamination of CIAV was at 0.1 EID_50_ per 1000 feathers_,_ the reproducibility of qPCR was poor and the CT value for the contaminated vaccines was very close to the negative samples.

In this study, the ddPCR method established for the detection of CIAV contamination in vaccines confirmed the best detection range and conditions, which also showed a higher sensitivity than conventional PCR or qPCR methods. Contamination of CIAV in vaccines at 0.1 EID_50_/1,000 feathers could be directly detected. Therefore, ddPCR has great practical significance for detecting contamination in vaccines; the use of it could help monitor the vaccine quality and then reduce the outbreak of CIA.

## Figures and Tables

**Table 1:** Primers and probes used to amplify the sequence.

Application	Name	Sequences	Product size (bp)
PCR/ Real-time PCR/ ddPCR	CIAV-F	GCAGGGGCAAGTAATTTCAA	164
	CIAV-R	GCCACACAGCGATAGAGTGA	
	Probe	ACTGCAGAGAGATCCGGATTGGTATCG	

**Table 2:** Sensitivity and specificity of PCR, qPCR, and ddPCR.

Detection	CIAV standard plasmids (copies/*μ*L)				NDV	IBV	FPV
	10^3^	10^2^	10^1^	1			
PCR	+	+	-	-	-	-	-
qPCR	+	+	+	-	-	-	-
ddPCR	+	+	+	+	-	-	-

Note: “+” means positive results and “-” means negative results.

**Table 3:** Repeatability and CV value of ddPCR using standard recombinant plasmid.

Template quantity (copy)	Measured value	Average value	Standard deviation	Coefficient of variation%
2.4×10^0^	1.3	1.57	0.05	3.18
	1.8			
	1.6			

2.4×10^1^	18.1	18.03	0.21	1.16
	18.2			
	17.8			

2.4×10^2^	235	236.7	2.08	0.87
	239			
	236			

2.4×10^3^	2960	3010	104.4	3.45
	2940			
	3130			

**Table 4:** Comparison of the sensitivity of different methods for detecting the contamination of CIAV in NDV vaccine.

Detection	Vaccines with different concentrations of CIAV (EID_50_)			
	10^2^	10^1^	10^0^	10^−1^
Conventional PCR	+	+	-	-
qPCR	+	+	+	-
ddPCR	+	+	+	+

Note: “+” means positive results and “-” means negative results.

**Table 5:** Repeatability and CV value of ddPCR in the detection of CIAV contamination in vaccine.

Sample	Copies	Average	Standard deviation	CV%
0.1 EID_50_	0.31	0.4166667	0.929157	22%
	0.46			
	0.48			
1 EID_50_	6.8	6.566667	2.516611	3.83%
	6.3			
	6.6			
10 EID_50_	88.5	90.26667	15.94783	1.77%
	90.7			
	91.6			
100 EID_50_	933	940.6667	215.484	2.29%
	965			
	924			

## Data Availability

The data used to support the findings of this study are included within the article.
